# The RNA m^6^A Reader YTHDF2 Is Essential for the Post-transcriptional Regulation of the Maternal Transcriptome and Oocyte Competence

**DOI:** 10.1016/j.molcel.2017.08.003

**Published:** 2017-09-21

**Authors:** Ivayla Ivanova, Christian Much, Monica Di Giacomo, Chiara Azzi, Marcos Morgan, Pedro N. Moreira, Jack Monahan, Claudia Carrieri, Anton J. Enright, Dónal O’Carroll

**Affiliations:** 1 MRC Centre for Regenerative Medicine, Institute for Stem Cell Research, School of Biological Sciences, University of Edinburgh, 5 Little France Drive, Edinburgh EH16 4UU, UK; 2 European Molecular Biology Laboratory (EMBL), Mouse Biology Unit, Via Ramarini 32, Monterotondo Scalo 00015, Italy; 3 European Molecular Biology Laboratory (EMBL), European Bioinformatics Institute (EBI), Wellcome Genome Campus, Hinxton, Cambridge CB10 1SD, UK

**Keywords:** YTHDF2, m^6^A-reader, m^6^A, maternal transcriptome, oocyte maturation

## Abstract

YTHDF2 binds and destabilizes N^6^-methyladenosine (m^6^A)-modified mRNA. The extent to which this branch of m^6^A RNA-regulatory pathway functions *in vivo* and contributes to mammalian development remains unknown. Here we find that YTHDF2 deficiency is partially permissive in mice and results in female-specific infertility. Using conditional mutagenesis, we demonstrate that YTHDF2 is autonomously required within the germline to produce MII oocytes that are competent to sustain early zygotic development. Oocyte maturation is associated with a wave of maternal RNA degradation, and the resulting relative changes to the MII transcriptome are integral to oocyte quality. The loss of YTHDF2 results in the failure to regulate transcript dosage of a cohort of genes during oocyte maturation, with enrichment observed for the YTHDF2-binding consensus and evidence of m^6^A in these upregulated genes. In summary, the m^6^A-reader YTHDF2 is an intrinsic determinant of mammalian oocyte competence and early zygotic development.

## Highlights

• YTHDF2 deficiency is partially permissive in mice and required for female fertility
• YTHDF2 is maternally required for early zygotic development
• YTHDF2 post-transcriptionally regulates transcript dosage during oocyte maturation
• Maternal YTHDF2 is a key determinant of mammalian egg quality

## Graphical Abstract

**Figure:**
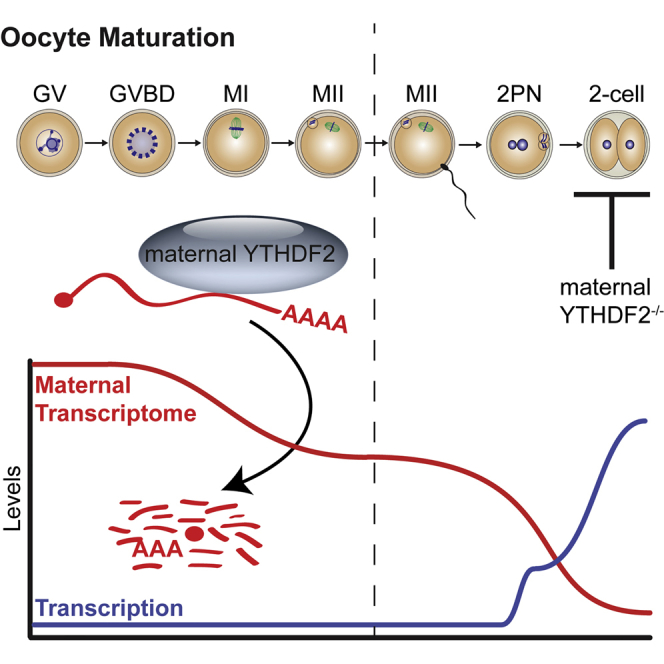

## Teaser

In this issue of *Molecular cell*, Ivanova et al. report an *in vivo* function of the RNA m^6^A reader YTHDF2 in the regulation of the maternal transcriptome during oocyte maturation and its importance for the establishment of competent oocytes that can sustain early embryo development.

## Introduction

RNA N^6^-methyladenosine (m^6^A) is the most abundant internal mRNA modification (Desrosiers et al., 1974, Perry, 1974) that is a key determinant of post-transcriptional mRNA regulation, with proven functions in mRNA processing, translation, and degradation (Fu et al., 2014). m^6^A is found enriched within the METTL3/14 methyltransferase RRACH (where R = G/A and H = A/C/U) consensus in the 3′UTR near the stop codon (Csepanys et al., 1990, Dominissini et al., 2012, Liu et al., 2014, Meyer et al., 2012, Ping et al., 2014, Schwartz et al., 2013, Wang et al., 2014b). The outcome of RNA methylation is instructed through changes in tertiary structure that recruit or displace defined RNA-binding proteins (Alarcón et al., 2015, Liu et al., 2015b) or by directly increasing the affinity of the binding site for YTH-domain-containing proteins (Theler et al., 2014). The mouse genome encodes five YTH domain-containing proteins, one nuclear (YTHDC1) (Xiao et al., 2016, Xu et al., 2014) and four normally cytoplasmic (YTHDF1–YTHDF 3 and YTHDC2) (Shi et al., 2017, Tanabe et al., 2016, Wang et al., 2014a, Wang et al., 2015). YTHDC1 regulates splicing and nuclear export (Xiao et al., 2016). While YTHDF1/3 recruitment enhances translation (Shi et al., 2017, Wang et al., 2015), the binding of YTHDF2 to m^6^A mRNA within the GACU/A consensus is associated with its destabilization and degradation (Du et al., 2016, Wang et al., 2014a). YTHDF2 is required for early zebrafish embryonic development (Zhao et al., 2017). RNA m^6^A is required for mouse embryonic stem cell exit from pluripotency and preimplantation development (Batista et al., 2014, Geula et al., 2015, Wang et al., 2014b); however, the *in vivo* and developmental contribution of the m^6^A mRNA-YTHDF2-mediated mRNA destabilization pathway in mammals remains unknown.

Transcription and translation are uncoupled during defined stages of gametogenesis and early zygotic development; thus the regulation of gene expression occurs at the post-transcriptional level. In spermatogenesis, the lepto-zygotene stages of meiotic prophase as well as the latter part of spermiogenesis are transcriptionally inert (Monesi, 1964, Paronetto et al., 2011). Indeed, ALKBH5, an m^6^A RNA demethylase, is required for normal mouse spermatogenesis (Zheng et al., 2013). The maternal transcriptome is assembled during the growth phase of oogenesis and is completed with the cessation of transcription in full-grown prophase I germinal vesicle (GV) oocytes (Eppig and Schroeder, 1989). Post-transcriptional regulation and utilization of the maternal transcriptome underpin meiotic maturation, fertilization, and early embryonic development (Bachvarova et al., 1985, Paynton et al., 1988). Oocyte maturation is hormonally triggered and occurs just prior to ovulation when GV oocytes complete meiosis I and advance to metaphase II (MII) (Li and Albertini, 2013). In mice, these ovulated MII oocytes enter the oviduct, where they await fertilization. Oocyte maturation is accompanied by a wave of RNA degradation where approximately 20% of total maternal RNA is actively degraded (Bachvarova et al., 1985, Ma et al., 2013, Paynton et al., 1988, Su et al., 2007). This absolute reduction in cellular mRNA results in relative changes to transcript dosage in the MII transcriptome, where some transcripts are stabilized, are destabilized, or remain unchanged (Ma et al., 2013, Su et al., 2007). The MII transcriptome is a large determinant of oocyte competence (Li et al., 2010), and the mechanisms ensuring correct gene dosage achieved through meiotic maturation are not known.

## Results

### YTHDF2 Is a Cytoplasmic Protein Expressed at All Stages of Mammalian Gametogenesis

**Figure 1:**
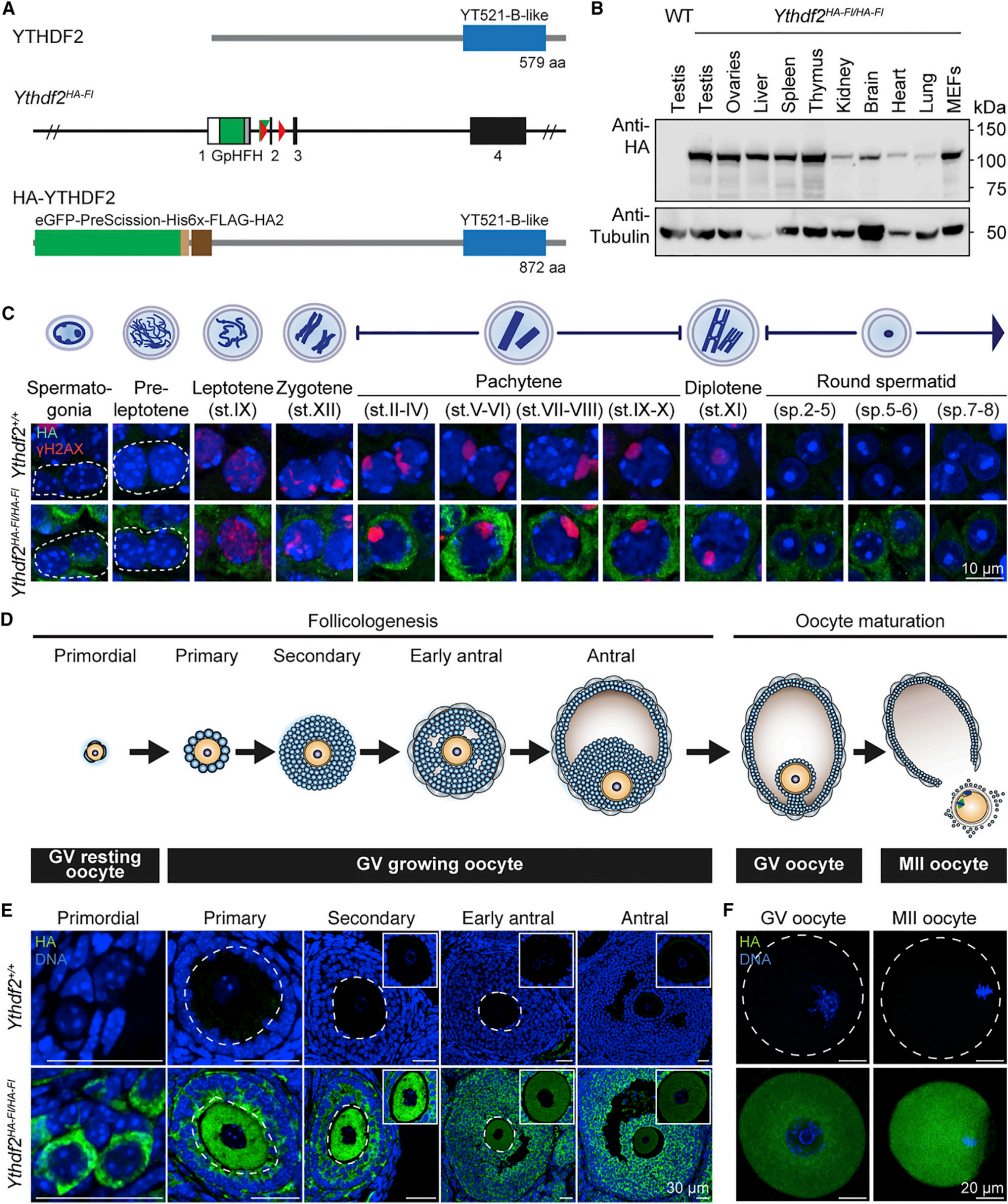
The RNA m^6^A Reader YTHDF2 Is Expressed in Multiple Tissues, in Mouse Fibroblasts, and in the Germline (A) Schematic representation of the YTHDF2 protein, the *Ythdf2*^*HA-Fl*^ allele, and the HA-YTHDF2 fusion protein. (B) Western blot using anti-HA and anti-α-tubulin antibodies on the indicated tissue and cell line lysates from *Ythdf2*^*HA-Fl/HA-Fl*^ and wild-type (WT). (C) Top panel is a schematic representation of spermatogenesis. Below are shown confocal immunofluorescent images of testis sections stained with anti-HA antibody (green) and γ-H2AX (red) of the indicated spermatogenic cells from *Ythdf2*^*+/+*^ and *Ythdf2*^*HA-Fl/HA-Fl*^ mice. Scale bar, 10 μm. (D) Schematic representation of folliculogenesis and oocyte maturation. Abbreviations: GV, germinal vesicle oocyte; MII, metaphase II-arrested oocyte. (E) Confocal immunofluorescent images of *Ythdf2*^*+/+*^ and *Ythdf2*^*HA-Fl/HA-Fl*^ ovary sections stained with anti-HA antibody (green) and Hoechst (blue) for primordial, primary, secondary, early antral, and antral follicular stages. Scale bar, 30 μm. Top right corner square is a magnified image of the oocyte in the respective follicle. (F) Confocal immunofluorescence images of GV and MII oocytes of wild-type and homozygous *Ythdf2*^*HA-Fl*^ mice stained with anti-HA antibody (green) and Hoechst (blue) are shown as indicated. Scale bar, 20 μm. See also Figure S1.

To understand the *in vivo* function of YTHDF2 and m^6^A-mediated mRNA destabilization of transcripts, we generated an epitope-tagged and conditional allele of *Ythdf2* (*Ythdf2*^*HA-Fl*^*)* in mice (Figures 1A, S1A, and S1B). The N-terminal tagging of YTHDF2 with GFP-His6-FLAG-HA did not affect the function of the protein, as mice homozygous for *Ythdf2*^*HA-Fl*^ were viable and fertile (Figure S1C, related to Figure 1). The HA-YTHDF2 protein was detectable by western blotting and was expressed in all tissues analyzed (Figure 1B). However, the respective tissues express differential amounts of YTHDF2, with testis displaying the highest expression (Figure 1B). We next sought to understand the expression of YTHDF2 at the cellular resolution in the germline during gametogenesis. YTHDF2 is expressed at all stages of spermatogenesis, with elevated expression observed in pachytene spermatocytes (Figures 1C). Folliculogenesis is the growth phase of oocyte development where the biomaterial and the maternal transcriptome required for oocyte competence are assembled (Eppig and Schroeder, 1989). This initiates when a clutch of primordial oocytes commence growth coincident with the expansion of surrounding somatic granulosa cells that collectively form the follicles with folliculogenesis, culminating in ovulation (Figure 1D) (Matzuk et al., 2002). At all stages of folliculogenesis, YTHDF2 is expressed both in the oocyte and in somatic granulosa cells (Figure 1E). YTHDF2 is also expressed during oocyte maturation, with abundant YTHDF2 detected in GV as well as in MII oocytes (Figure 1F). Both during spermatogenesis and folliculogenesis, YTHDF2 is cytoplasmic in both the germ and the somatic cells (Figures 1C, 1E, and 1F).

### *Ythdf2* Deficiency Is Partially Permissive in Mice and Results in Female-Specific Infertility

**Figure 2:**
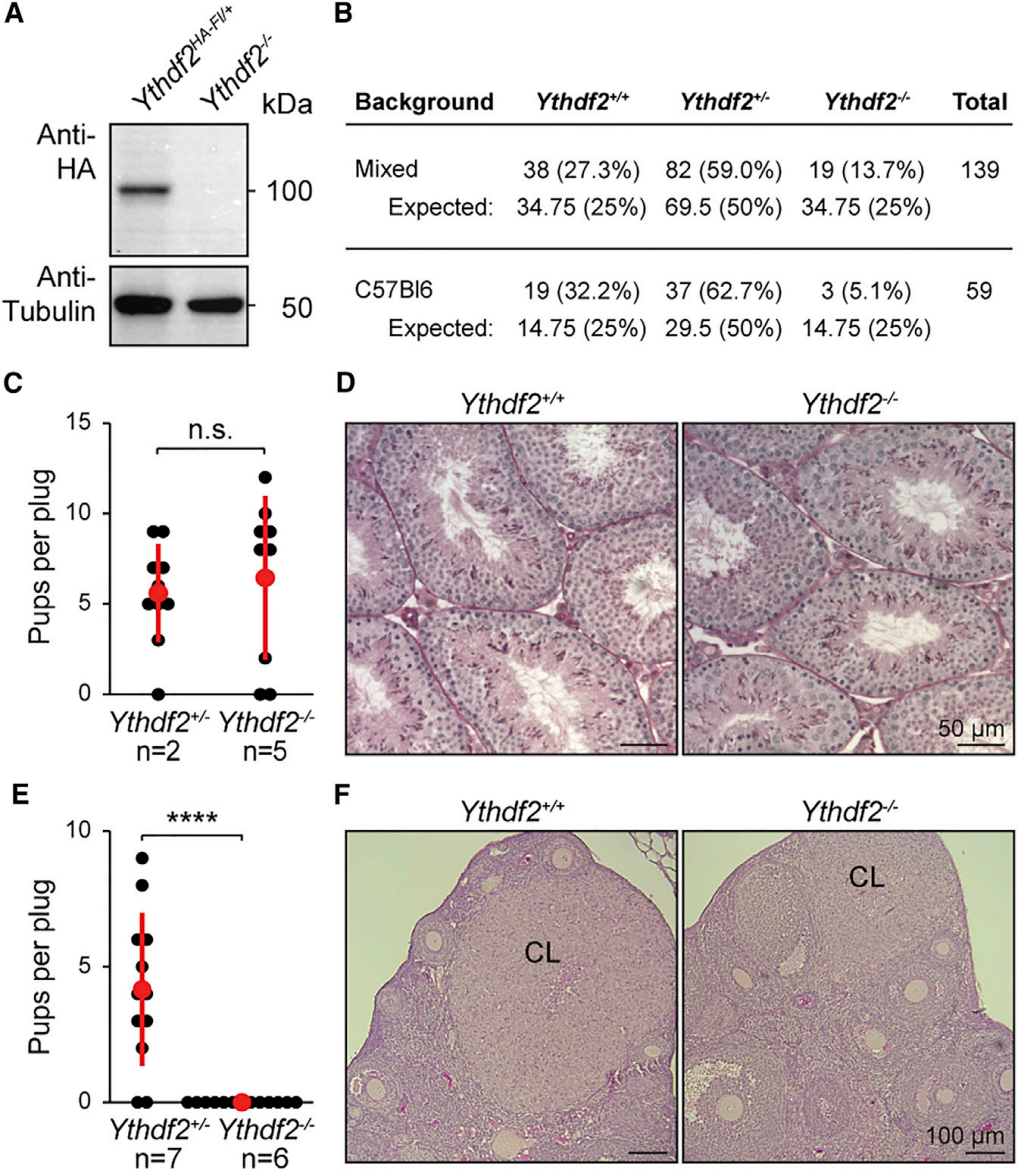
YTHDF2 Is Required for Female Fertility (A) Western blot using anti-HA and anti-α-tubulin antibodies on testis lysates from *Ythdf2*^*HA-Fl/+*^ and *Ythdf2*^*−/−*^. (B) Table of the numbers and percentages of pups at weaning and the expected Mendelian numbers of animals per genotype from *Ythdf2*^*+/−*^ intercrosses from mixed and C57Bl6 genetic background. (C) The number of pups born per plug from *Ythdf2*^*+/−*^ and *Ythdf2*^*−/−*^ male mice is shown. The number (n) of animals tested, the mean and SD are indicated (t test; n.s., p > 0.05). (D) Representative PAS stained testis section from wild-type and *Ythdf2*^*−/−*^ mice. Scale bar, 50 μm. (E) The number of pups born per plug from *Ythdf2*^*+/−*^ and *Ythdf2*^*−/−*^ female mice is shown. The number (n) of animals tested, the mean, and SD are indicated (t test; ^∗∗∗∗^p < 0.00001). (F) Representative PAS-stained ovary section from wild-type and *Ythdf2*^*−/−*^ mice. CL indicates corpus luteum. Scale bar, 100 μm. See also Figure S1.

To understand the *in vivo* function of YTHDF2, we converted the conditional allele to a null allele (*Ythdf2*^*−*^) (Figures 2A, S1A, and S1B). We observed that *Ythdf2*^*−/−*^ mice are retrieved in sub-Mendelian ratios (Figure 2B), with approximately half of the expected *Ythdf2*^*−/−*^ mice observed at weaning. The loss of *Ythdf2*^*−/−*^ from heterozygous intercrosses increased as the allele was bred toward a C57Bl6 genetic background (Figure 2B). Nonetheless, the viable *Ythdf2*^*−/−*^ mice were indistinguishable from their heterozygous or wild-type littermates. We next determined the fertility status of both male and female *Ythdf2*^*−/−*^ mice by set-up crosses with wild-type mice. Despite the abundant YTHDF2 expression throughout spermatogenesis (Figure 1C), *Ythdf2*^*−/−*^ males were fertile (Figure 2C), with normal seminiferous tubule histology (Figure 2D). However, *Ythdf2*^*−/−*^ females were sterile (Figure 2E), with corpora lutea observed in the *Ythdf2*^*−/−*^ ovaries, indicating that ovulation had occurred (Figure 2F). In summary, YTHDF2 deletion is partially permissive in mice and results in female-specific infertility.

### YTHDF2 Is Intrinsically and Maternally Required for Oocyte Competence

**Figure 3:**
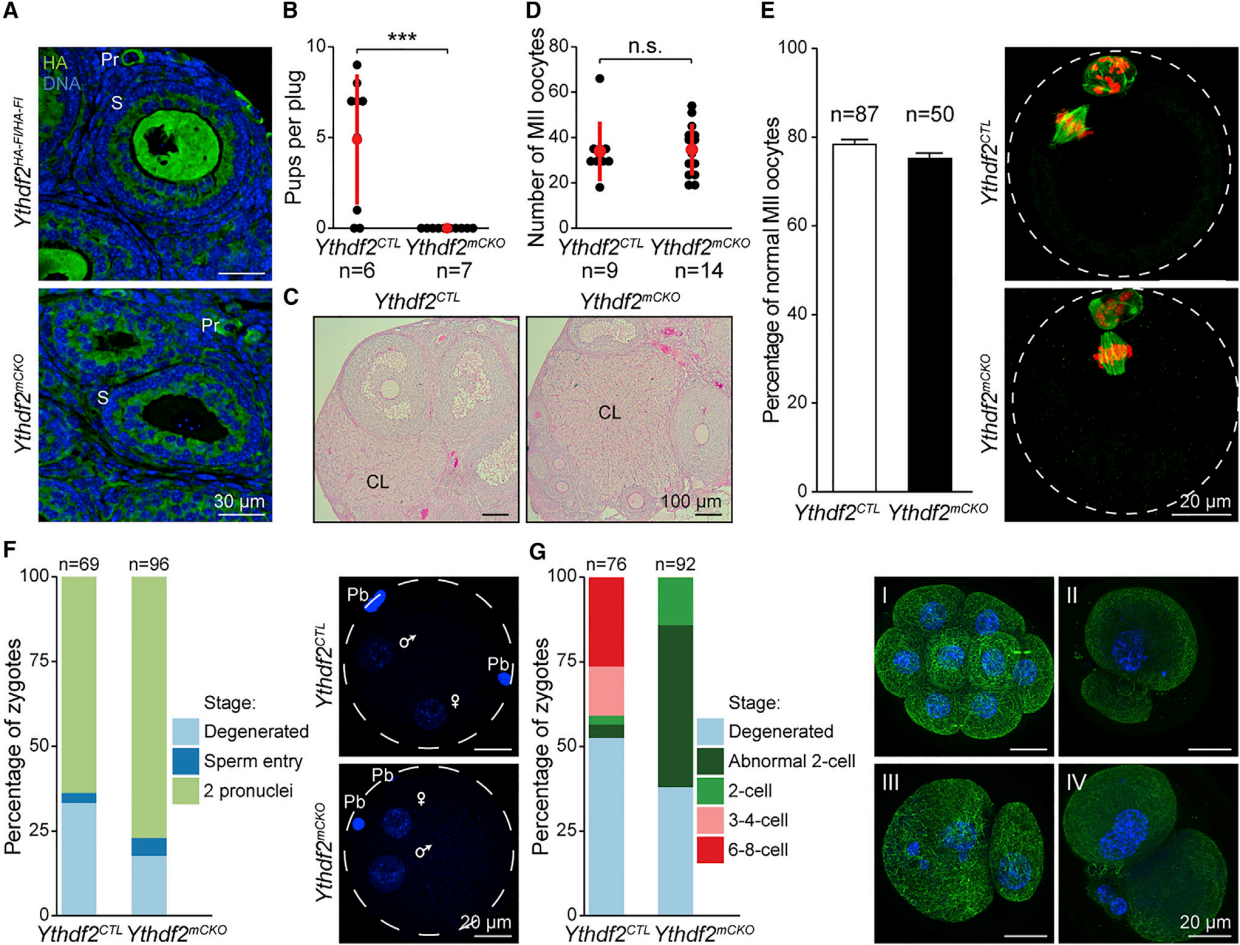
YTHDF2 Is Maternally Required for Oocyte Maturation and Early Zygotic Development (A) Confocal immunofluorescence with anti-HA antibody (green) and Hoechst (blue) on ovary sections from homozygous *Ythdf2*^*HA-Fl*^ and *Ythdf2*^*mCKO*^ mice are shown. Primordial (Pr) and secondary (S) follicle stage are indicated. Scale bar, 30 μm. (B) Number of pups born per plug from *Ythdf2*^*CTL*^ and *Ythdf2*^*mCKO*^ female mice. The number (n) of animals, mean, and SD are indicated (t test;^∗∗∗^p < 0.0001). (C) PAS-stained ovary sections from *Ythdf2*^*CTL*^ and *Ythdf2*^*mCKO*^ females. CL indicates corpus luteum. Scale bar, 100 μm. (D) Number of MII oocytes isolated from oviduct of hormone-primed *Ythdf2*^*CTL*^ and *Ythdf2*^*mCKO*^ female mice. The number (n) of animals, the mean, and SD are indicated (t test; n.s., p > 0.05). (E) Percentage of normal MII oocytes isolated from hormone-primed *Ythdf2*^*CTL*^ and *Ythdf2*^*mCKO*^ females (n indicates number of oocytes). The morphology of MII oocytes was assessed through immunofluorescent staining with anti-β-tubulin antibody (green) and Hoechst (blue). Representative images for *Ythdf2*^*CTL*^ and *Ythdf2*^*mCKO*^ MII oocytes are shown, with the zona pellucida indicated by a white dashed circle. Scale bar, 20 μm. (F) Percentage of degenerated, sperm entry, and two pronuclei zygotes isolated from hormone-primed and stud male-mated *Ythdf2*^*CTL*^ and *Ythdf2*^*mCKO*^ female mice at embryonic day 0.5. The number (n) of zygotes assessed is indicated. Confocal immunofluorescent images of representative *Ythdf2*^*CTL*^ and *Ythdf2*^*mCKO*^ zygotes stained with Hoechst (blue) are shown, with the zona pellucida indicated by a white dashed circle. The female and male pronuclei are indicated with female and male signs, respectively; Pb denotes polar bodies. Scale bar, 20 μm. (G) Percentage of degenerated, abnormal two-cell, two-cell, three- to four-cell, and six- to eight-cell zygotes isolated from stud male-mated hormone-primed *Ythdf2*^*CTL*^ and *Ythdf2*^*mCKO*^ female mice at embryonic day 2.5. The number of zygotes assessed is indicated. Confocal immunofluorescent images of representative *Ythdf2*^*CTL*^ 8-cell zygote (Panel I) and *Ythdf2*^*mCKO*^ arrested abnormal two-cell zygotes (Panels II–IV) stained with anti-β-tubulin (green) and Hoechst (blue) are shown. Scale bar, 20 μm.

The female infertility in *Ythdf2*^*−/−*^ mice could arise from either germline- or somatic-related defects. Indeed, YTHDF2 is expressed both in the oocyte and in somatic granulosa cells during folliculogenesis (Figure 1E). The somatic cells support and transmit key instructive signals to the growing oocyte (Li and Albertini, 2013). We therefore employed conditional genetics to test the intrinsic and maternal oocyte function of YTHDF2. To this end, we combined the *Zp3Cre* allele that deletes in growing oocytes with our *Ythdf2*^*HA-Fl*^ allele to generate experimental *Ythdf2*^*HA-Fl/HA-Fl*^*; Zp3Cre Tg*^*+*^ (*Ythdf2*^*mCKO*^) and control *Ythdf2*^*HA-Fl/+*^*; Zp3Cre Tg*^*+*^ or *Ythdf2*^*+/+*^*; Zp3Cre Tg*^*+*^ (*Ythdf2*^*CTL*^) mice. This maternal conditional deletion (mCKO) strategy resulted in the oocyte-specific deletion of YTHDF2 without affecting its expression in somatic granulosa cells (Figure 3A). Crossing of *Ythdf2*^*mCKO*^ females with wild-type males revealed that maternal expression of YTHDF2 is intrinsically required for female fertility (Figure 3B). Histological analysis of *Ythdf2*^*mCKO*^ ovaries revealed the presence of corpora lutea, indicating that ovulation has occurred (Figure 3C). Hormone priming with pregnant mare serum gonadotropin (PMSG) and human chorionic gonadotropin (hCG) to induce oocyte growth and subsequent ovulation revealed that *Ythdf2*^*mCKO*^ females produce normal numbers of MII oocytes that have completed meiosis I and arrested at metaphase II properly (Figures 3D and 3E). We next sought to understand if *Ythdf2*^*mCKO*^ oocytes are competent to be fertilized. *Ythdf2*^*CTL*^ and *Ythdf2*^*mCKO*^ females were hormone primed and set up with wild-type males, and zygotes were collected 0.5 days later and examined for fertilization as evidenced by the progression to the 2 pronuclei (2PN) stage. A similar frequency of *Ythdf2*^*mCKO*^ and *Ythdf2*^*CTL*^ oocytes had reached the 2PN stage accompanied by the extrusion of the second polar body (Figure 3F); thus YTHDF2 is not required for the process of fertilization per se. Harvesting zygotes at 2.5 days after priming and mating revealed that development is derailed at or prior to the two-cell stage in *Ythdf2*^*mCKO*^ zygotes (Figure 3G). In comparison to *Ythdf2*^*CTL*^, fewer *Ythdf2*^*mCKO*^ zygotes made two-cell stage embryos of normal morphology, with many of the *Ythdf2*^*mCKO*^ two-cell embryos presenting various cytokinesis defects such as micronuclei and enucleated cells (Figure 3G). In summary, YTHDF2 is maternally required for early zygotic mouse development.

### YTHDF2 Post-transcriptionally Regulates Transcript Dosage during Meiotic Maturation

**Figure 4:**
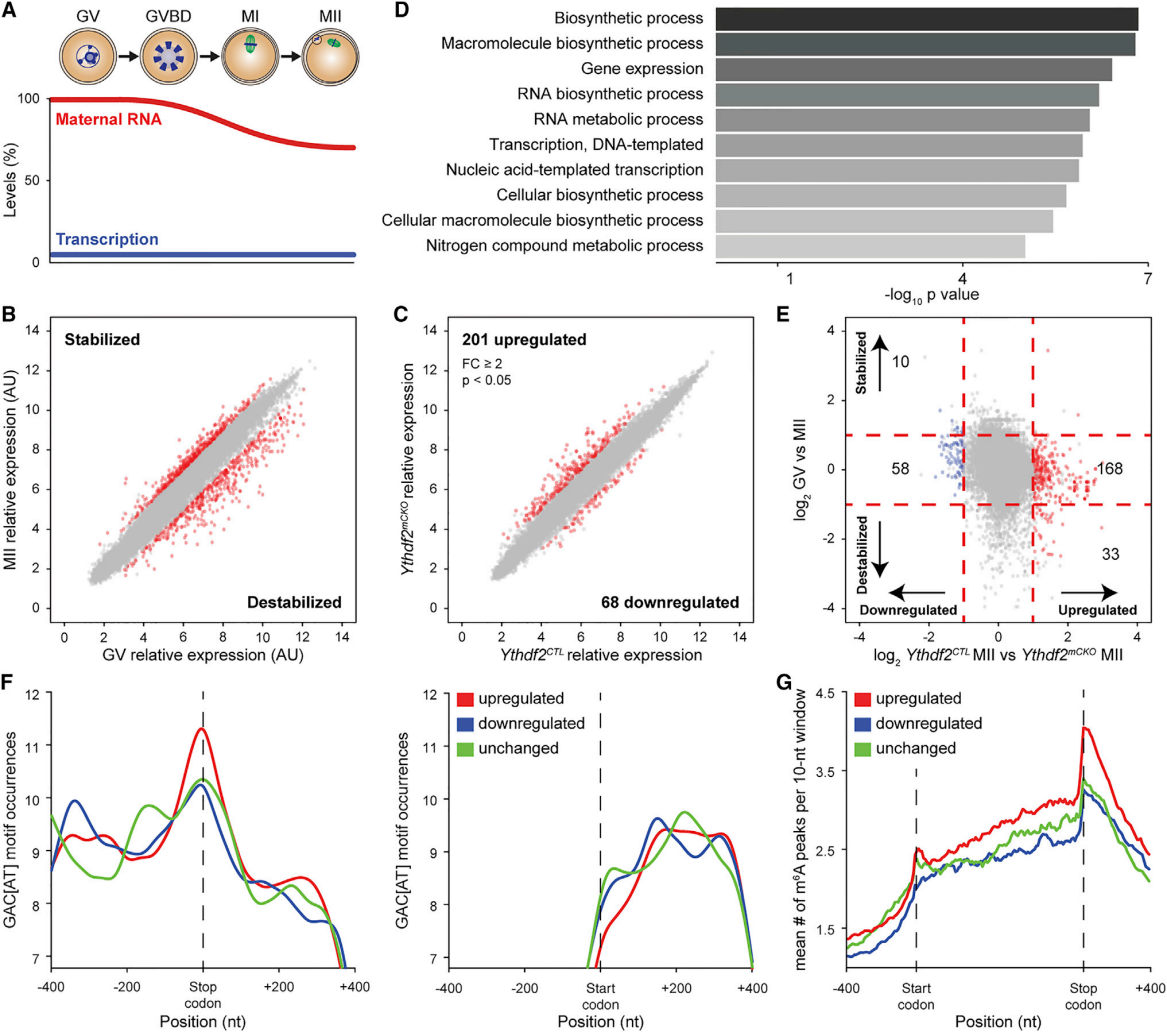
YTHDF2 Regulates Maternal Transcript Dosage during Oocyte Maturation (A) Schematic representation of oocyte maturation with a graph indicating the approximate levels of maternal RNA and transcription. Abbreviations: GV, germinal vesicle oocyte; GVBD, germinal vesicle break down oocyte; MI, metaphase I oocyte; and MII, metaphase II-arrested oocyte. (B) Expression scatterplot showing the relative average expression of transcripts from GV and MII oocytes. In red are highlighted the genes that are significantly changed (p ≤ 0.05) with a fold difference greater or equal to 2 between GV and MII oocytes. Analysis was done on biological three to four replicas. (C) Expression scatterplot showing the relative average expression of transcripts between *Ythdf2*^*CTL*^ and *Ythdf2*^*mCKO*^ MII oocytes. Significantly deregulated (p < 0.05) genes with a fold change greater than or equal to 2 are shown in red. Analysis was done on biological triplicates. (D) Gene ontology analysis for the upregulated genes in *Ythdf2*^*mCKO*^ MII oocytes; the top ten most significant processes identified are shown. (E) Expression scatterplot showing the relationship between transcriptome changes during oocyte maturation (shown on the y axis) and changes in *Ythdf2*^*mCKO*^ with respect to *Ythdf2*^*CTL*^ MII oocytes (shown on the x axis). The significantly upregulated and downregulated genes in *Ythdf2*^*mCKO*^ MII oocytes are shown in red and blue points, respectively. The unchanged transcripts in oocyte maturation and in *Ythdf2*^*CTL*^ versus *Ythdf2*^*mCKO*^ MII oocytes are indicated by horizontal and vertical dashed red lines, respectively. The number of genes in the respective gates is indicated. (F) Graph representation of Loess smoothed sum of YTHDF2-binding motif GACU/A occurrences ±400 nt around the stop codon (left panel) and around the start codon (right panel). Data are shown for the top 1,000 most-upregulated transcripts (red), 1,000 downregulated transcripts (blue), and 1,000 transcripts whose expression remained unchanged (green) in *Ythdf2*^*mCKO*^ MII oocytes. Only enrichment of the upregulated genes compared to unchanged genes around the stop codon is statistically significant (p < 0.05). (G) Prevalence of m^6^A peaks from public mouse datasets around the gene bodies of upregulated, downregulated, and unchanged transcripts in *Ythdf2*^*mCKO*^ MII oocytes. Gene bodies are scaled to the same length in each case. Only enrichment of the upregulated genes compared to unchanged genes around the stop codon is statistically significant (p < 0.05). See also Figures S2, S3, and S4.

The failure of *Ythdf2*^*mCKO*^ oocytes to support early zygotic development could arise from the inability to process or utilize the transcriptome correctly. The utilization of the maternal transcriptome commences with the onset of oocyte maturation with an overall 20% reduction of cellular RNA in MII oocytes that results in relative changes to the MII transcriptome (Figures 4A and 4B) (Bachvarova et al., 1985, Flemr et al., 2010, Su et al., 2007). We decided to analyze the MII transcriptome, as this is the earliest stage where the maternal transcriptome is utilized, thus enabling us to determine the primary impact of YTHDF2 on the maternal transcriptome. Analysis of MII oocytes revealed deregulated gene expression within the *Ythdf2*^*mCKO*^ transcriptome, with increased dosage of transcripts originating from 201 genes and decreased expression from 68 loci when applying a cut-off with a fold change greater or equal to two and a significance with a p value less than 0.05 (Figures 4C and S2). To exclude a function for YTHDF2 in the formation of the maternal transcriptome, we profiled gene expression in GV oocytes where transcription has ceased and the oocyte possesses a mature maternal transcriptome (Bachvarova et al., 1985, Paynton et al., 1988). This analysis revealed that *Ythdf2*^*mCKO*^ GV oocytes contain a near normal transcriptome (Figure S3, related to Figure 4). In summary, the loss of YTHDF2 does not grossly impact oocyte growth or the formation of the maternal transcriptome but is required to instruct the correct gene dosage during oocyte maturation. The bias in deregulated gene expression in *Ythdf2*^*mCKO*^ MII oocytes toward upregulation is consistent with the removal of a protein that potentiates RNA degradation. We next sought to understand what class of genes YTHDF2 regulates across oocyte maturation, whether those that are relatively stabilized, destabilized, or remain unchanged (Figure 4E). This analysis revealed that in the cohort of genes that are upregulated in *Ythdf2*^*mCKO*^ MII oocytes, the majority (168/201) should remain relatively unchanged across oocyte maturation, and some (33/201) are destabilized (Figures 4E and S4). In summary, YTHDF2 function is intrinsically and maternally required to instruct the appropriate transcript dosage during oocyte maturation.

We next sought to understand if some of the upregulated transcripts could be direct targets of YTHDF2. GACU/A is the most common YTHDF2-binding motif identified from PAR-CLIP (Wang et al., 2014a). We therefore looked for enrichment of the GACU/A consensus ±400 bp around the stop codon in the top upregulated and downregulated genes as well as in genes whose dosage remains unchanged. To this end, we took the top 1,000 upregulated and downregulated transcripts; this selection is required for statistical power and corresponds to an approximate 1.4-fold change in expression levels. This analysis revealed a significant enrichment for the GACU/A motif only in upregulated genes in *Ythdf2*^*mCKO*^ MII oocytes (Figure 4F). This enrichment was not observed around the 5′UTR (Figure 4F), where YTHDF2 is known not to occupy under steady-state non-stress conditions (Wang et al., 2014a). The limiting amount of RNA that can be isolated from oocytes excludes the possibility of performing m^6^A-seq to determine if the consensus-containing upregulated genes in *Ythdf2*^*mCKO*^ MII oocytes are methylated. We therefore sought to determine if we can find evidence for their methylation in other mouse tissues using the MeT-DB database (Liu et al., 2015a). We found a stark enrichment for m^6^A adjacent to the stop codon in the transcripts that are upregulated in *Ythdf2*^*mCKO*^ MII oocytes (Figure 4G). In summary, we find that the upregulated transcripts in the *Ythdf2*^*mCKO*^ MII oocytes are enriched for the m^6^A/YTHDF2 consensus motif, and for some we find evidence for their m^6^A methylation in other cell types.

## Discussion

The loss of METTL3 demonstrated an indispensable function for RNA m^6^A in embryonic stem cell exit from pluripotency and preimplantation development (Batista et al., 2014, Geula et al., 2015, Wang et al., 2014b). Here we report the deletion of a mammalian m^6^A reader and identify the physiological importance of the m^6^A-YTHDF2-mediated mRNA destabilization pathway in mice. We identify and characterize an essential role for YTHDF2 in the female germline. We also find that loss of YTHDF2 is partially permissive in mice, indicating that YTHDF2 has other important developmental functions outside of the germline. The presence of *Ythdf2*^*−/−*^ viable adult mice indicates that this branch of the RNA m^6^A regulatory pathway is not essential for mice under normal conditions. However, given that YTHDF2 has been shown to be involved in the cellular heat-shock response (Zhou et al., 2015), it remains to be seen if YTHDF2 function is required *in vivo* to appropriately respond to various physiological and environmental perturbations. The fact that *Ythdf2*^*−/−*^ mice show a genetic background effect indicates that mRNA m^6^A sites could constitute a basis for modifier alleles. If this is the case, it would demonstrate the power of mRNA m^6^A sites as potential modifiers of development and disease. This could be especially relevant to human development and disease given the increased frequency of RNA m^6^A observed in human versus mouse cell lines (Dominissini et al., 2012). While the mouse m^6^A demethylase ALKBH5 is required for normal spermatogenesis (Zheng et al., 2013), we demonstrate that the YTHDF2-mediated destabilization of m^6^A-containing transcripts is dispensable for male gametogenesis, at least on a mixed genetic background. In yeast RNA, m^6^A is specific to meiosis (Clancy et al., 2002); the loss of Ime4, an ortholog of METTL3, or the YTH-domain containing m^6^A reader Mrb1, affects meiotic progression (Schwartz et al., 2013, Shah and Clancy, 1992). In mice, YTHDF2 does not regulate the dosage of meiosis-specific genes (Figure 4D), with meiosis I and likely II being completed normally (Figure 3E) in *Ythdf2*^*mCKO*^ oocytes. *Ythdf2*^*−/−*^ male mice are fertile, with no impact observed in spermatogenesis (Figures 2C and 2D). In summary, the YTHDF2-mediated regulatory RNA m^6^A pathway is not important for mouse meiosis.

The maternal loss of zebrafish Ythdf2 has a modest impact on zygotic development, whereas maternal or zygotic Ythdf2 depletion profoundly impairs embryogenesis (Zhao et al., 2017). Here we show a defining maternal function for YTHDF2 in regulating transcript dosage across oocyte maturation which is essential for generating MII oocytes that are competent to sustain early zygotic development. The loss of YTHDF2 during oocyte maturation results in the deregulation of approximately 270 genes, leading to an arrest prior to or at the two-cell stage, with various cytokinesis defects observed in the two-cell embryos (Figure 3G). The maternal transcriptome is essential for the first mitotic division (Clift and Schuh, 2013). We posit that the deregulated gene expression in *Ythdf2*^*mCKO*^ oocytes poisons the maternal transcriptome, rendering it incompetent to support the mitotic division. We have shown that YTHDF2 is expressed throughout oocyte growth and from GV through to MII oocytes but that the phenotype only arises after meiotic resumption (Figures 3E–3G). Furthermore, YTHDF2 does not majorly impact on the formation of the maternal transcriptome (Figure S3, related to Figure 4), although, present throughout oocyte growth, these observations beg the question as to why YTHDF2-mediated mRNA degradation is only active upon meiotic maturation. In somatic cells, YTHDF2 has been shown to function through the recruitment of deadenylases and subsequent mRNA decapping (Du et al., 2016, Wang et al., 2014a). RNA degradation factors are downregulated during oocyte growth, favoring the accumulation of RNA and the building of the maternal transcriptome (Flemr et al., 2010). During meiotic maturation, the deadenlyase CNOT7 and the decapping enzymes DCP1A and DCP2 are translated from maternal transcripts that enable the resumption of active RNA degradation pathways (Ma et al., 2013, Ma et al., 2015). We believe this reactivation of RNA degradation machinery enables YTHDF2 to directly degrade the bound transcripts selectively during meiotic maturation. In summary, we demonstrate that the m^6^A reader YTHDF2 is an essential regulator of the mammalian maternal transcriptome and egg quality.

## STAR★Methods

### Key Resources Table

**Table:** 

REAGENT or RESOURCE	SOURCE	IDENTIFIER
**Antibodies**		

Mouse monoclonal HA.11 clone 16B12 antibody	previously Covance	MMS-101P
Mouse monoclonal anti-α-tubulin	Sigma-Aldrich	T9026
Mouse monoclonal anti-β-tubulin antibody	Sigma-Aldrich	T4026
Rabbit polyclonal anti-γH2AX	ICH	ICH-00059
Mouse IgG HRP-linked antibody	Amersham ECL	NA931

**Chemicals, Peptides, and Recombinant Proteins**		

Amersham Hybond-XL membrane	GE Healthcare	RPN203S
Immobilon-P membrane	Millipore	IPVH00010
Pregnant mare serum gonadotropin (PMSG)	Henry Schein	N/A
Human chorionic gonadotropin (hCG)	Intervet	N/A
Hyaluronidase	Sigma-Aldrich	H3884
M2 media	Sigma-Aldrich	M7167
Hoechst33342	Sigma-Aldrich	14533
Antigen unmasking solution	Vector Lab	H-3300
Normal donkey serum	Sigma-Aldrich	D9663
Teflon-coated slides	Dutscher scientific	6110016
Bouin’s solution	Sigma-Aldrich	HT10132
Schiff reagent	Sigma-Aldrich	s5133
Hematoxylin	Sigma-Aldrich	H3136

**Critical Commercial Assays**		

QIAzol lysis reagent	QIAGEN	79306
Ovation Pico WTA Systems	NuGEN	N/A
Encore Biotin Module	NuGEN	N/A
GeneChip Mouse Gene 2.0 ST Array	Affymetrix	N/A

**Deposited Data**		

Expression data	This paper	E-MTAB-5056
Expression data	This paper	E-MTAB-5576
Mendeley Data dataset	This paper	http://dx.doi.org/10.17632/zb7zyfghg3.1

**Experimental Models: Organisms/Strains**		

Mouse: ZP3Cre:C57Bl6-Tg	(de Vries et al., 2000)	N/A
Mouse: Ythdf2^HA-Fl^	This paper	N/A
Mouse: Ythdf2^-^	This paper	N/A

**Oligonucleotides**		

Primers for qRT-PCR, see Table S1		

**Software and Algorithms**		

Limma	(Ritchie et al., 2015)	https://bioconductor.org/biocLite.R
Gorilla	(Eden et al., 2009)	http://cbl-gorilla.cs.technion.ac.il
Biomart	(Smedley et al., 2015)	http://www.biomart.org
Smooth.spline	R/Bioconductor	https://rdrr.io/bioc/aroma.light/src/R/likelihood.smooth.spline.R
DeepTools package v1.5.9.1	(Ramírez et al., 2014)	http://deeptools.readthedocs.io/en/2.0.1/source/deeptools.html

### Contact for Reagent and Resource Table

Further information and requests for resources and reagents should be directed to and will be fulfilled by the Lead Contact, Dónal O’Carroll (donal.ocarroll@ed.ac.uk).

### Method Details

#### Generation of alleles and mice in this study

For the *Ythdf2*^*HA-Fl*^ allele, a GFP-precission-His6-Flag-HA-HA epitope tag was inserted after the endogenous starting initiation ATG codon in exon 1 of *Ythdf2*. In addition, two *loxP* sites were placed flanking exon 2. The targeting construct was genetically modified so that it contained homology arms and FRT sites flanking a neomycin cassette 3′ of exon 1. Southern blotting of EcoRV-digested DNA extracted from ESC-derived clones with exogenous 5′probe was used for the validation of homologous recombinants. The wild-type *Ythdf2* locus generates a ∼9 kb DNA fragment, whereas the integration of the second *loxP* site introduced an additional EcoRV site, thus decreasing the size of the EcoRV DNA fragment to 8 kb in the targeted allele. Flp-mediated recombination removed the FRT flanked neomycin cassette and generated the *Ythdf2*^*HA-Fl*^ allele that can be identified by the 5′ probe as a 6 kb EcoRV DNA fragment. Cre-mediated deletion of the *loxP* flanked exon 2 resulted in 5.5 kb EcoRV DNA fragment, that can be identified by the 5′probe and validate the *Ythdf2*^*-*^ allele. The *Zp3Cre Tg* (de Vries et al., 2000) allele was also used in this study for the generation of *Ythdf2*^*mCKO*^ female mice. Fertility analysis with male and female *Ythdf2*^*−/−*^ mice were performed on a mixed genetic background. The majority of the *Ythdf2*^*mCKO*^ analysis was performed on mice that were backcrossed six times toward the C57BL/6 genetic background. All mice used in this study were on mixed or C57BL/6 genetic background and were bred and maintained in EMBL Mouse Biology Unit, Monterotondo, and subsequently at the Centre for Regenerative Medicine, Edinburgh. All procedures were done in accordance to the current Italian legislation (Art. 9, 27. Jan 1992, nu116) under license from the Italian health ministry or the UK Home Office regulations, respectively.

#### Southern blotting

The forward 5′-GCAGGTGACCTCTTCAGAAG-3′ and reverse 3′-CCAGTCCCTGTAGATTTTAGAG-5′ primers were used to generated an exogenous 5′ probe for detection of the targeted, *Ythdf2*^*HA-Fl*^ and *Ythdf2*^*-*^ alleles. Genomic DNA was restriction digested and run on a 0.8% agarose gel. The DNA fragments were then transferred to an Amersham Hybond-XL membrane (GE Healthcare RPN203S) through alkaline solution (0.4 M NaOH, 1.5 M NaCl) overnight. The membrane was neutralized in 2X SSC solution, UV-crosslinked with 150 mJ/cm^2^ and incubated in prehybridization solution (0.5 M Na_2_HPO_4_, 1 mM EDTA, 5% SDS, 3% BSA) for 2 hr at 65°C. DNA probe was synthesized with Random Primers DNA Labeling System (Thermo Fisher Scientific) in accordance to manufacturer’s protocol and was hybridized with the membrane overnight at 65°C. The membrane was washed in 40 mM Na_2_HPO_4_, 1 mM EDTA, 5% SDS and exposed on a phosphor screen (Fujifilm).

#### Western blotting

Protein extracts were prepared with dounce homogenizer in lysis buffer (50 mM Tris pH 7.8, 150 mM NaCl, 0.4% NP-40, 2 mM MgCl_2_, 1 mM DTT) supplemented with proteinase inhibitors. Extracts were collected after centrifugation at 14000 g for 10 min at 4°C, resolved on 7.5% SDS-PAGE gel and transferred on Immobilon-P membrane (Millipore) via wet transfer overnight. Membranes were blocked in 5% milk-PBST (PBS with 0.1% Tween20) and probed with anti-HA (Covance, MMS-101P, 1:1000) and anti-alpha-tubulin antibody (Sigma-Aldrich, T9026, 1:1000) for 4 hr at room temperature. Membrane was washed in PBST and incubated with appropriate horseradish peroxidase-coupled secondary antibody (Amersham) in 5% milk-PBST for 1 hr. Proteins were detected with ECL Western Blotting Detection Reagent (Amersham) and acquired on a ChemiDoc XRS system (BioRad).

#### Oocyte and zygote collection

For the collection of GV oocytes, 3-8 weeks old females were injected with 10 U of pregnant mare serum gonadotropin (PMSG) (Henry Schein). After 44-48 hr GV oocytes were collected through puncturing the ovarian follicles in M2 media (Sigma-Aldrich). Subsequently, GV oocytes were released from the somatic cells via manual mechanical separation.

For the collection of MII oocytes, 3-8 weeks old females were injected with 10 U of PMSG and after 46-48 hr with 10 U of human chorionic gonadotropin (hCG) (Intervet). MII oocytes were isolated from the oviduct of the hormone-stimulated females 14 hr after the hCG injection. MII oocytes were cleaned from the somatic cells with hyaluronidase (Sigma-Aldrich) in M2 media.

For the collection of zygotes, PMSG and hCG stimulated females (as described above) were set up with a stud male immediately after the last injection. Zygotes were isolated from the oviduct of plugged females 0.5 and 2.5 days after the hCG injection. The collected 0.5 day zygotes were briefly cleaned from the somatic cells with hyaluronidase in M2 media.

#### Immunofluorescence

For the detection of YTHDF2 in GV and MII oocytes, the isolated oocytes of the respective genotypes were stained with mouse anti-HA antibody (Covance, MMS-101P, 1:100) and Hoechst33342 (5 mg/ml) (Sigma-Aldrich,) as previously described (Dietrich et al., 2015).

For immunofluorescence on ovary sections, ovaries were dissected and fixed in 4% paraformaldehyde for 2 hr at 4°C and subsequently sucrose-OCT embedded. Ovary sections of 6 μm were cut on cryostat and subjected to antigen retrieval with antigen unmasking solution (Vector Lab H-3300). Next, sections were permeabilized with 0.1% Triton-X for 15 min at room temperature (RT). Blocking was performed with 10% normal donkey serum (NDS) (Sigma-Aldrich, D9663), 2% BSA and 0.1 M glycine for 30 min at RT. Sections were incubated with anti-HA mouse antibody (1:100) for 1 hr at 37°C in a humidified chamber. Appropriate secondary antibodies from Invitrogen were applied for 1 hr at RT, followed by Hoechst 33342 for DNA staining.

For the microtubule staining, MII oocytes and zygotes were fixed in 2% formaldehyde, SB solution (0.01 M Pipes, 0.5 mM MgCl_2_, 0.25 mM EGTA) and treated with 2% Triton-X for 30 min at 37°C. The MII oocytes/zygotes were washed three times in 0.1% NDS and transferred to blocking solution (10% NDS and 0.01% Triton-X) for 1 hr. Then MII oocytes/zygotes were incubated with rabbit anti-beta tubulin antibody (Sigma-Aldrich) (1:200) at 37°C for 1 hr. They were washed three times in the blocking solution before incubation with secondary antibody in 5% NDS and Hoechst 33342 staining. MII oocytes/zygotes were mounted on teflon coated slides (Dutscher scientific) in a group of 10 per well.

Immunofluorescence assay on testis sections was performed as previously described (Comazzetto et al., 2014). The above mouse anti-HA antibody and anti-γH2AX (ICH) (1:250) rabbit antibodies were used.

Images were acquired on SP5 and SP8 Leica TSC confocal microscope. The max projection images were cropped and modified in Photoshop with equal settings for control and experimental samples.

#### Histology

Ovaries and testis from adult mice were fixed overnight in Bouin’s solution and embedded in paraffin. Sections of 7 μm were cut and stained with periodic acid Schiff reagent (Sigma-Aldrich) and Hematoxylin (Sigma-Aldrich) as per manufacturer’s instructions.

#### Oocytes mRNA expression analysis

Total RNA was isolated from 50-90 GV and MII oocytes with QIAzol lysis reagent (QIAGEN) following the manufacturer instructions. Total RNA was *in vitro* transcribed and biotinylated with the Ovation Pico WTA Systems V2 (NuGEN) and fragmented and labeled with Encore Biotin Module (NuGen). Hybridization of the cDNA was done with GeneChip Mouse Gene 2.0 ST Array (Affymetrix) for 16 hr at 45°C. Affymetrix Fluidics Station 450 was used for washes and staining of the GeneChips. For this analysis 3-4 biological replicates were used for GV and MII *Ythdf2*^*CTL*^ and *Ythdf2*^*mCKO*^ oocytes.

#### Statistics

For RNA expression analysis, robust multi-array average (RMA) was used for the raw data normalization and limma package to determine differential expression (Ritchie et al., 2015). Moderate t-statistics was done with adjusted p values. Gene ontology analysis was done with Gene ontology enrichment analysis and visualization tool Gorilla (Eden et al., 2009).

#### Motif analysis

Affymetrix identifiers were mapped to Ensembl transcripts using Biomart (*32*). Biomart was then used to obtain 400 nt 5′ and 3′ of both the start and stop codon. Sequences obtained that were shorter than 400 nt were padded to this length with the addition of ’N’s. Each transcript may only be present once in the Affymetrix gene list. Where multiple transcripts are present, the one with the highest absolute fold-change is retained. Only one isoform is retained when multiple transcripts have the same sequence. Motif occurrences were directly computed using Perl regular expression matching for “GCA[UA]” for sequences assigned to the top 1000 most upregulated, downregulated and for 1000 transcripts from the center of the gene list. A matrix of 1000x800 elements is hence obtained for each of the three sets, indicating which sequence and at which nucleotides motifs occur. This matrix is column summed and plotted using a cubic spline smoothing function smooth.spline from R/Bioconductor. Statistics for the motif enrichment was done with a hypergeometric test.

#### m^6^A peaks dataset analysis

Public m^6^A peak data was obtained from MeT-DB (http://compgenomics.utsa.edu/methylation/) for 12 mouse (mm9) samples. Peaks were translated into BED files with enrichment scores, lifted-over to mm10 via UCSC liftover, sorted, filtered and overlapping regions condensed into bigwig files. These bigwig files were provided to the DeepTools package v1.5.9.1 (ComputeMatrix and Heatmapper) (Ramírez et al., 2014) to explore peak enrichments around gene-bodies from the start to the stop codon with 400 nt either side. The Ensembl GTF corresponding to GRCm38 version 79 was used for gene body coordinates. Statistics for the m^6^A peak enrichment was done with two-sample t test.

#### qRT-PCR

Total RNA from 50-80 MII oocytes per biological replicate was reverse-transcribed using SuperScript IV and random hexamers (both Invitrogen) according to manufacturer’s instructions. qRT-PCR was performed using the LightCycler 480 SYBR Green I Master mix (Roche), and samples were run in technical triplicates on a Roche LightCycler 480 instrument. C_t_ values were normalized against the internal controls *Gapdh*, *Sod1* and *Bmp15*. Fold differences in expression levels were calculated according to the 2^−ΔΔCT^ method.

### Data and Software Availability

Original images, immunoblots and radiography can be found in Mendeley Data (http://dx.doi.org/10.17632/zb7zyfghg3.1).

## Author Contributions

I.I. contributed to the design, execution, and analysis of most of the experiments. D.O. conceived this study. C.M. designed, generated, and validated the *Ythdf2*^*HA-Fl*^ allele and qRT-PCR experiments. M.D.G. and C.A. contributed to histology and imaging experiments. I.I., C.C., J.M., and M.M. performed the bioinformatic transcriptome analysis. P.N.M. aided in analysis of mouse zygotes. A.J.E. performed the consensus enrichment analysis and oversaw all bioinformatic analyses performed. D.O. and A.J.E. supervised this study. I.I., A.J.E., and D.O. wrote the final version of the manuscript.

## Supplemental Information

Document S1. Figures S1–S4 and Table S1

Document S2. Article plus Supplemental Information
