# Supplementary material for: The RNA m6A Reader YTHDF2 Is Essential for the Post-transcriptional Regulation of the Maternal Transcriptome and Oocyte Competence
Source: Mol Cell. 2017 Sep 21;67(6):1059–1067.e4. doi: 10.1016/j.molcel.2017.08.003 (PMC5613143; doi:10.1016/j.molcel.2017.08.003)
Supplement: Document S1. Figures S1–S4 and Table S1 [file mmc1.pdf]

Molecular Cell, Volume *67*

Supplemental Information

The RNA m^{6}A Reader YTHDF2 Is Essential for the Post-transcriptional Regulation of the Maternal Transcriptome and Oocyte Competence

Ivayla Ivanova, Christian Much, Monica Di Giacomo, Chiara Azzi, Marcos Morgan, Pedro N. Moreira, Jack Monahan, Claudia Carrieri, Anton J. Enright, and Dónal O'Carroll

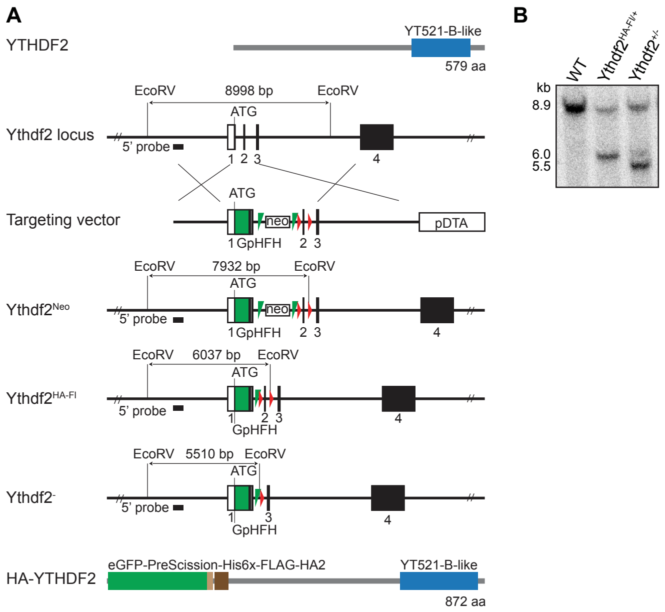

C

Background	Wild type	*Ythdf2*$^{HA-Fl/+}$	*Ythdf2*$^{HA-Fl/HA-Fl}$	Total
Mixed	26 (23.2%)	56 (50.0%)	30 (26.8%)	112
Expected:	28 (25%)	56 (50%)	28 (25%)	

Figure S1. Generation and validation of the *Ythdf2*$^{HA-Fl}$ allele (related to Figure 1 and Figure 2)

(A) Targeting strategy for the generation of the *Ythdf2*$^{HA-Fl}$ allele. The domain structure of YTHDF2 is presented (top). The blue rectangle represents the YT521-B-like domain that binds to the RNA m^{6}A modification. The 5' part of the *Ythdf2* locus and the targeting vector used to insert GFP-PreScission-His6-Flag-HA2 after the starting ATG codon including two loxP sites (illustrated by red rectangles) surrounding exon 2 are shown. FRT sites flanking the neomycin (neo) resistance cassette are illustrated by green rectangles. The targeted (*Ythdf2*^{Neo}), *Ythdf2*$^{HA-Fl}$ and *Ythdf2*$^{-}$ alleles are also shown with the EcoRV restriction enzyme sites and the respective size of the fragments generated for the Southern blot validation of the respective alleles. The position of the 5' probe used in the Southern blot hybridization is shown as black rectangle. At the bottom is the domain structure of the YTHDF2 protein with the N-terminal tag.

(B) Southern blot of the EcoRV restriction enzyme generated fragments from wild type, *Ythdf2*$^{HA-Fl}$ and *Ythdf2*$^{+/-}$ tail DNA, hybridized with the 5' probe is shown.

(C) Table of the numbers and percentages of pups at weaning and the expected Mendelian numbers of animals per genotype from *Ythdf2*$^{HA-Fl/+}$ intercrosses from mixed genetic background is shown.

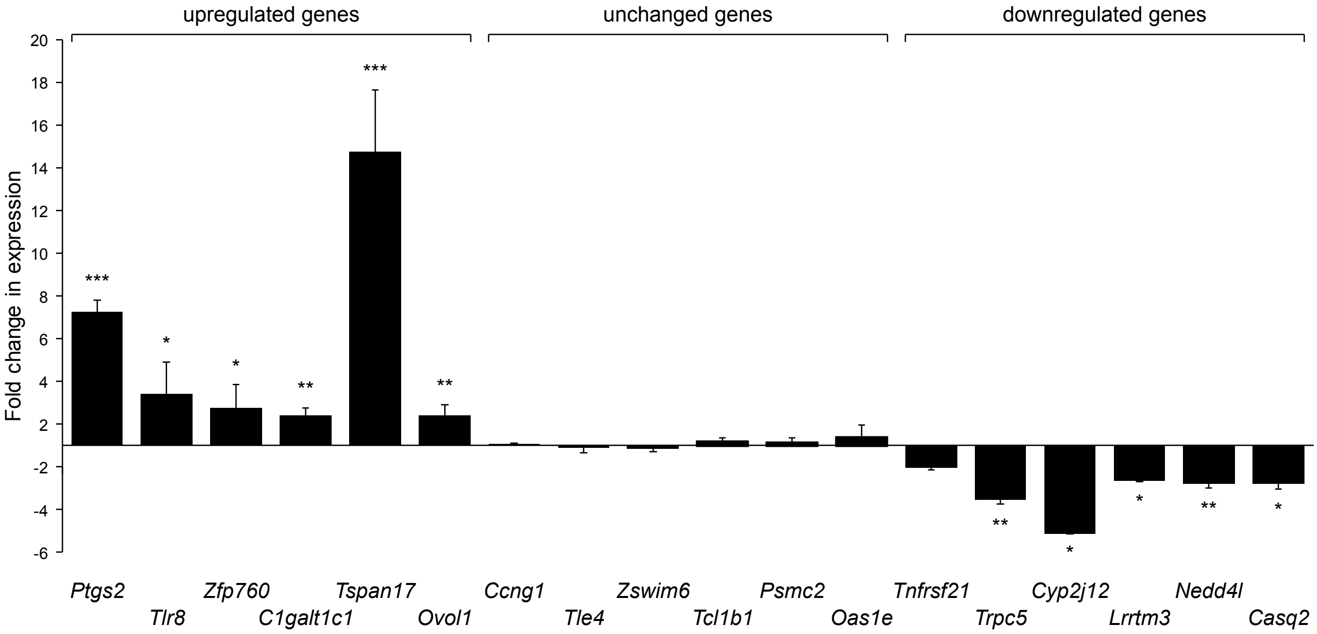

Figure S2. Validation of gene expression changes in YTHDF2-deficient MII oocytes (related to Figure 4)

Fold change in gene expression in *Ythdf2*^{mCKO} MII oocytes relative to *Ythdf2*^{CTL} MII oocytes as determined by qRT-PCR is shown. Depicted are representative genes that have been determined to be upregulated (also representing transcripts with m^{6}A peaks from MeT-DB analysis), unchanged and downregulated according to microarray profiling. C_t values were normalized against *Gapdh*, *Sod1* and *Bmp15*. Biological triplicates are shown; the bars' heights and vertical lines indicate the mean and s.d., respectively. Significance is indicated (t-test; *, $p < 0.05$; **, $p < 0.01$; ***, $p < 0.001$).

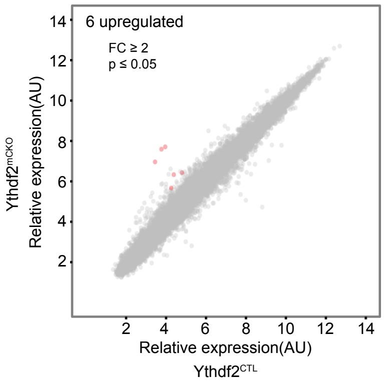

Figure S3. Oocyte-specific deletion of *Ythdf2* has little effect on the transcriptome of GV oocytes (related to Figure 4)

Expression scatterplot represents the relative average expression of transcripts between *Ythdf2*^{CTL} and *Ythdf2*^{mCKO} GV oocytes. Significantly deregulated ($p < 0.05$) genes with a fold change greater than 2 are shown in red.

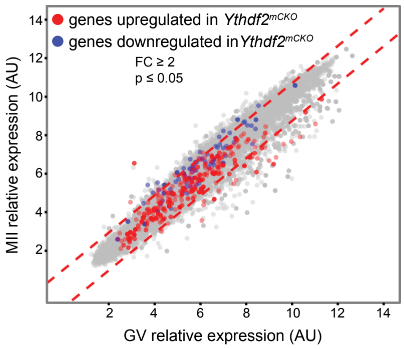

Figure S4. YTHDF2 regulates transcript dosage in the GV to MII oocyte transition (related to Figure 4)
Expression scatter plot showing the relative behavior of the upregulated (red points) and downregulated (blue points) genes in *Ythdf2^{mCKO}* MII oocytes compared to the relative average expression of transcripts in GV and MII oocytes. Red dashed lines separate the unchanged fraction of transcripts between GV and MII oocytes, genes above or below are stabilized and destabilized in MII oocytes, respectively.

Gene	Forward	Reverse
Gapdh	TGTCGTGGAGTCTACTGGTG	TTCACACCCATCACAAACAT
Sod1	CGGTGAACCAGTTGTGTTGT	GGTCTCCAACATGCCTCTCT
Ptgs2	CCAGCACTTCACCCATCAGT	GGGGATACACCTCTCCACCA
Tlr8	AGCCTTCCAAGAAAGAATTTGGG	CAGCAGACAAAAGCACGTCAG
Zfp760	AACAGGAAGAAATGGCTGCTT	AGACCTTCTCATATTTACCACACAT
C1galt1c1	AGCGTAACAGAGTGGGCCTT	GGTGGTGCATTCTGTTTCCAA
Tspan17	ATGTCAAAGCCTACCGGGAT	TCTTCCGCTGGGTCCCTAAC
Ovol1	TTCCTGGTGAAGAAGCCATGC	TCGAAGGCTCATGTCCAAAGC
CCng1	TGGCTTTGACACGGAGACAT	GCTCAGTCCAACACACCCAA
Tle4	GAGCAAGATGTACCCGCAGA	CTGGCGAGCTTCTCACATTC
Zswim6	TGGAGAGTGGCTGCGTAGA	GGTTTCTGGCTCCGGTTGTA
Tcl1b1	GGTCTCCGTTAGACTGGGC	AGGGAATAACCTGCTGGGGG
Psmc2	GAGGCACTTGCGGCTTCTAA	GGCCGTAAGTTTTCAGCAAGG
Oas1e	GTGAATGGCTGGGAAGTGTC	TCAAAGTCTTACAGCGGAGG
Tnfrsf21	TGATGGAAGACACCACGCAG	CACTTGTTCTTGTCCAGCGG
Trpc5	TGAACTCCCTCTACCTGGCA	GCCCTAAATGGGAGTTGGCT
Cyp2j12	ACCCAAGAACTACCCACCAG	GTCCAGGCTAGTTAGGTTTCC
Lrrtm3	TAGCAAATCAGGCTCCAGGG	GGTGGCTTTCCATCGTGTCT
Nedd4l	GACCAGCCTTCCTCTCCCT	CACTTTGGGTTCAGCGTCTT
Casq2	GCGGTGGCAAAGAAGTTATCC	CCCATTCAAGTCGTCTTCCCA

Table S1. qRT-PCR primers (related to Method details and the Key Resource Table)
